# The Effect of *Ilex × meserveae* S. Y. Hu Extract and Its Fractions on Renal Morphology in Rats Fed with Normal and High-Cholesterol Diet

**DOI:** 10.3390/foods10040818

**Published:** 2021-04-09

**Authors:** Piotr Kuropka, Anna Zwyrzykowska-Wodzińska, Robert Kupczyński, Maciej Włodarczyk, Antoni Szumny, Renata M. Nowaczyk

**Affiliations:** 1 Department of Biostructure and Animal Physiology, Division of Histology and Embryology, Wroclaw University of Environmental and Life Sciences, Norwida 25, 50-375 Wroclaw, Poland; piotr.kuropka@upwr.edu.pl; 2 Department of Environment Hygiene and Animal Welfare, Wroclaw University of Environmental and Life Sciences, Chelmońskiego 38C, 51-631 Wroclaw, Poland; anna.zwyrzykowska@upwr.edu.pl (A.Z.-W.); robert.kupczyński@upwr.edu.pl (R.K.); 3 Department of Pharmacognosy and Herbal Medicines, Faculty of Pharmacy, Wroclaw Medical University, Borowska 211a, 50-556 Wroclaw, Poland; maciej.wlodarczyk@umed.wroc.pl; 4 Department of Chemistry, Wroclaw University of Environmental and Life Sciences, Norwida 25, 50-375 Wroclaw, Poland; antoni.szumny@upwr.edu.pl

**Keywords:** *Ilex × meserveae*, Yerba Mate, kidney filtration barrier, high-cholesterol diet, saponins, terpenoids, polyphenols

## Abstract

Therapeutic properties of *Ilex* species are widely used in natural medicine. *Ilex × meserveae* may become a potential substitute for *Ilex paraguariensis* (Yerba Mate). As a part of the preliminary safety verification of this European *Ilex* hybrid vs. Yerba Mate, an eight-week study concerning the impact of regular administration of leaves of both species on kidneys was conducted. The standard water infusion and three dominant fractions of *Ilex × meserveae* leaves’ constituents (polyphenols, saponins and less polar terpenoids) were separately tried on 96 male Wistar rats divided into 8-member groups. Animals were divided into two basic nutritional groups: the first one was rats fed standard feed and the second on was rats fed with high-cholesterol diet (20 g of cholesterol per kg of standard feed). Postmortem morphometric evaluation of stained kidney samples concerned the filtration barrier elements, which are crucial in proper diuresis. The results showed that saponins present in the hydroalcoholic dry extract (administered in a dose of 10 mg/kg of body weight/day) as well as in water infusions (1:20) from *Ilex × meserveae* and *Ilex paraguariensis* do not demonstrate nephrotoxicity but conversely, have a protective role on kidney status in animals fed with a normal diet and in a high-cholesterol diet.

## 1. Introduction

*Ilex* L. species, especially *Ilex paraguariensis* A. St.-Hil., are frequently used in traditional medicine. The brew (infusion) of *Ilex paraguariensis* leaves, known as Yerba Mate, was ritually used by Native South Americans before the colonizers’ arrival. This beverage’s consumption is recently being expanded to many North America, Asia, and European countries. In trade, Yerba Mate, in its ground form, contains mainly leaves, together with fragments of young branches, flowers, and peduncles. Simultaneously, *I. paraguariensis* extracts are distributed as an additive in various products, e.g., cosmetics and food supplements as well as functional foods [1,2]. The research confirmed that the therapeutic effect of *Ilex* spp. concern arthritis, diabetes, immune diseases, hemorrhoids, headaches, liver dysfunction, and obesity [3]. Many studies have shown that obesity and related diseases are significant health problems [4,5]. In this plant’s case, the research indicated that *I. paraguariensis* water extract lowered total cholesterol and low-density lipoprotein in people with high levels of serum lipid, and thus is promiscuous to treat obesity [5,6], while hyperlipidemia can be a risk factor for the progression of renal diseases and changes of glomerular structure and thickness of basement membrane [7]. It is noteworthy that cholesterol modulates the bilayer structure parameters of biological membranes, such as thickness, compressibility, water penetration. However, high cholesterol and triglyceride plasma levels have been demonstrated to be important risk factors for the progression of kidney disease and high total cholesterol or reduced HDL (high-density lipoprotein) cholesterol can decrease glomerular filtration rate [8].

In terms of phytochemical research, *I. paraguariensis* has probably been the subject of the most intensive investigations among all *Ilex* spp. [3]. It should be noted that the content of active biological compounds present in *Ilex* species depends on extraction methods, phenotype, environmental variability, as well as harvest time [9]. Generally, *I. paraguariensis* extracts contain polyphenols (including flavonoids, tannins, chlorogenic acid, and its derivatives), purine alkaloids (methylxanthines; like caffeine or theobromine), vitamins (A, B, C, and E) as well as some triterpene saponins (derived mainly from ursolic acid) [10]. The main phenolic compounds in *I. paraguariensis* are associated with caffeoylquinic esters [11]. Our preliminary study [12] confirmed that *Ilex* species other than *I. paraguariensis* also contain a high amount of polyphenolic fraction (rich in rutin, quinic acid, and its caffeoyl esters), triterpenes, as well as their glycosides (saponins). The similarity of phytoconstituents in European *Ilex × meserveae* S. Y. Hu “Blue Angel” and South American *I. paraguariensis* allows us to consider *I. meserveae* “Blue Angel” as a promising source of bioactive compounds [12]. Of the above, caffeoylquinic esters were generally found to be interesting remedies for lowering blood cholesterol [5,6].

The *Ilex* secondary metabolites are mainly eliminated via the kidney route and impact the diuresis level due to dynamic changes in the glomerular filtration barrier (GFB) structure. The glomerular basement membrane provides stability for the filtration process and constitutes the GFB [13]. According to Jarad and Miner [14], the glomerular capillary wall consists of three layers: the glomerular basement membrane and the fenestrated endothelium, with its glycocalyx; the podocyte with interfoot processes and the fenestral diaphragm; and the glomerular basement membrane. These structures have been considered as the significant determinants of glomerular permeability with functional importance of two additional layers: the endothelial surface layer and the subpodocyte space; which all of the above structures have highly restrictive dimensions and contribute to the hydraulic resistance and ultrafiltration characteristics of the glomerulus [13,15]. Disturbed glomerular filtration barrier functions play a crucial role in developing many kidney diseases, including proteinuria [14,16].

Generally, it has been observed that drinking the infusion of many leaf extracts increases urination in animal models. There are only rare reports about the influence of *I. paraguariensis* beverages [17] on the urinary system, while no report on *I. meserveae* was found. However, the medical properties, including the impact on the high-cholesterol diet and also the side effects of *Ilex* spp., expected of *Ilex paraguariensis*, have not been studied in detail yet. Therefore, based upon our earlier study [12] on detailed characterization of polar, semipolar (polyphenolic, saponin), and less-polar (terpenoid) fractions from plants belonging to the *Ilex* genus, we decided to comparatively investigate the effects of *I. paraguariensis* and *I. meserveae* extract on kidney structure in animals fed with a normal and high-cholesterol diet. As factors modulating the impact of the adverse effects of cholesterol were used the extracts of *I. paraguariensis* (Yerba Mate) and *I. meserveae*, and fractions of the last one: polyphenols, terpenoids, and saponins.

## 2. Materials and Methods

### 2.1. Reagents and Plant Materials

The following solvents were used for UHPLC-MS (ultra-high-performance liquid chromatography coupled with mass spectrometry detector): acetonitrile (MS-purity; Sigma-Aldrich, St. Louis, MO, USA), water (LC-gradient; Merck, Kenilworth, NJ, USA), and formic acid (p.p.a. (pure per analysis), 98–100%, Merck). Analytically pure methanol (Chempur, Karlsruhe, Germany) and distilled water were used to extract *Ilex* leaves. Ballast substances were precipitated with lead (II) acetate (p.p.a, Chempur). The octadecyl bed for the SPE (solid-phase extraction) process was from J. T. Baker. Suitable solvents and reagents used for histological examinations were bought from Archem, Ommen, The Netherlands. 

The leaves of *Ilex* × *meserveae* S. Y. Hu “Blue Angel” (*Aquifoliaceae*, voucher Il.6/06.2016) were obtained from a nursery (Grodziszów, Poland) and authenticated by Professor Przemysław Bąbelewski. Immediately after harvesting, the leaves were frozen (−20 °C) and lyophilized to avoid enzymatic degradation of metabolites (20 h in 0.25 mBar, followed by 4 h in 0.025 mBar; Alpha 1–4 LDplus, Martin Christ). Directly after the lyophilization process, the leaves were reduced to a powder and extracted. Commercial Yerba Mate leaves (*Ilex paraguariensis* A. St.-Hil.), used as well-recognized standards in a saponin identification protocol, were purchased from two independent distributors (vouchers Il.1a/06.2016 and Il.1b/06.2016). Voucher specimens were deposited at the Department of Horticulture, The Faculty of Life Sciences and Technology, Wroclaw University of Environmental and Life Sciences, Wroclaw, Poland. 

### 2.2. Plant Extracts

#### 2.2.1. Water Extracts

Infusion of *I. paraguariensis and I. meserveae* were prepared day by day (across the animal experiment period) in the same manner by adding 50.0 g of dried and ground leaves to 1L of boiled water (80 °C), left for 20 min, then filtered. The typical process efficiency for *I. meserveae* was 1.4% (calc. on DM (dry mass)). Suspected active compounds in this extract were: simple sugars, polysaccharides, amino acids and other acids (including phenolic acids), small polar glycosides, polar polyphenols, saponins, and terpenoids listed in Appendix A (Table A1, Table A2 and Table A3).

#### 2.2.2. Polyphenols

Extracts from *I. meserveae* dried leaves were prepared with 80% methanol as the solvent, according to the method presented by Zwyrzykowska et al. [12]. The resulting fine powder was sufficiently soluble in water at experimental concentration. Process yield was 1.3% (calc. on DM). Detailed HPLC composition was published in [12].

#### 2.2.3. Saponins

Extracts from *I. meserveae* dried leaves were prepared and analyzed using a modified methodology developed by Włodaczyk et al. [18]. Briefly, the powdered plant material was cold-macerated with 70% methanol; the polar compounds (polysaccharides, peptides, phenolics) were precipitated and removed. Simultaneously, the supernatant was diluted and cleaned by solid-phase extraction (SPE) on an RP-18 bed to obtain a refined saponin fraction. This fraction was concentrated to dryness by consecutive vacuum evaporation and lyophilization. The resulting finely powdered dry extract fortified with saponins was sufficiently soluble in water at experimental concentrations. The yield of the saponin-enriched fraction was 2.5% (calc. on starting leaf DM). Suspected active compounds in this extract were mainly triterpenoid glycosides (saponins). Detailed isolation protocol and UHPLC-MS data are presented in Appendix A (Table A2 and Table A3).

#### 2.2.4. Terpenoids 

According to a protocol published in Polish Patent applications P.437122 and P.437123, the terpenoid fraction was obtained. The isolation details are presented in Appendix A.1.. Terpenoid fraction was dissolved in sunflower oil and added to animal feed. At the same time, the extract was monitored by gas chromatography with mass spectrometry (GC-MS). The yield of *I. meserveae* lipophilic fraction was 1.8% (calc. on starting DM). The detailed composition of the terpenoid fraction is presented in Appendix A (Table A1).

### 2.3. Animals, Housing, and Diets

All experimental procedures used in this study were approved by the II Local Ethics Committee in Wroclaw, Poland (permission No. 94/2015). The study was carried out on 96 male Wistar rats, aged 8 weeks, weight 250–280 g, kept in standardized environmental conditions (12/12 h light/dark cycle, the temperature approximately 22 °C, humidity about 55%). Every single cage counted two individuals. The animals were divided into two basic nutritional groups. The first main group (assigned with Roman numerals) was rats fed a standard feed and the second main group (assigned with Roman numerals followed by the letter a) was rats fed with a high-cholesterol diet (20 g of cholesterol per kg of standard feed). Each of the two main groups was divided into six subgroups consisted of 8 animals each. As factors modulating the impact of the negative effects of cholesterol were used the extracts of *I. paraguariensis* (Yerba Mate; **II**, **IIa**), *I. meserveae* (**III**, **IIIa**) and *I. meserveae* fractions: polyphenols (**IV**, **IVa**), terpenoids (**V**, **Va**) and saponins (**VI**, **VIa**). The subgroups **I** and **Ia** were the control groups (received no additional herbal extracts), while the other subgroups differed in the type of extracts added (data presented in Table 1). The doses of examined herbal extracts have been selected based on the de Resende et al. [19] study with our modification.

During the eight weeks of the experiment period, rats were fed ad libitum with a standard pelleted feed (composition: dry mass—906.04 (g/kg); energy content—19.78 (MJ/kg); total protein—17.42 (% DM); crude fat—2.13 (% DM); crude fiber—9.45 (% DM); Hybridpellet, Animalab, Poland). All animals had free access to drinking fluids, either water or *Ilex* infusion or *Ilex* fraction drink (250 mL/24 h/cage). Food and liquid ingestion and body weight (BW) were monitored daily throughout the experiment. At the end of the study, the animals were anesthetized with isoflurane and sacrificed by abdominal aorta exsanguinations. The kidneys were examined by the pathologist macroscopically in situ, based on the position, color, shape, size, and consistency of the organs.

### 2.4. Specimen Processing and Staining

The kidney samples were taken during the necropsy and immediately fixed in 10% neutral buffered formalin for three days, then washed in tap water for 24 h, dehydrated in a graded alcohol series, cleared in xylene, and finally embedded in paraffin. The 5 µm thick sections were routinely stained with hematoxylin and eosin (H&E, Sigma-Aldrich) and Alcian blue (Sigma-Aldrich) according to our modification. Histopathological observations were performed using a Nikon Eclipse 80i light microscope.

### 2.5. Statistical Analysis

Morphometric studies were carried out using the Nis-Elements Ar software (Nikon). A minimum of ten measurements of the glomerulus, the vascular loop, and the basement membrane’s thickness in the vascular loop was performed from each individual of the experimental group. The average was calculated based upon ten representative areas from the kidney cortex in each sample. All data were presented as mean ± SD. Statistical analysis was made using one-way analysis of variance (ANOVA) and performed using Statistica 6.0 (StatSoft), taking *p* < 0.05 as significant.

## 3. Results

### 3.1. Morphological Studies

Macroscopically, the kidneys’ structure, with a clear border between cortex and medulla, was well preserved in all experimental groups. The kidney capsule was not strongly anchored to the organ parenchyma. However, the kidneys’ edema and blood congestion were observed in some individuals of the group’s **II**, **IIa**, **IIIa**, **IVa**, **Va**, and **VIa**. The edema was mainly associated with increased diuresis.

### 3.2. Hematoxylin and Eosin Staining

**Figure 1:**
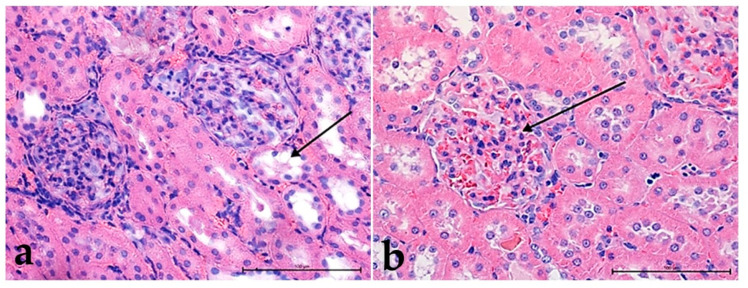
The structure of the kidneys in the control groups: in normal, **I** (**a**) and high-cholesterol diet, **II** (**b**); (**a**) note the lack of the urine in proximal tubules and slightly enlarged distal tubules (black arrow); (**b**) glomerulus filled with blood (black arrow) hematoxylin and eosin (H&E) staining. Mag. 400×. Bar scale 100 µm.

No significant changes were observed in the control group—**Ia** (Figure 1a). It should be noted that urogenesis and the transfer of urine through the tubules were slowed down. Proximal and distal tubules had enlarged light and were lined with a regular cuboidal epithelium (Figure 1a,b). In the case of the high-cholesterol control group—**Ib**, the renal glomeruli were congested, but there was no high urine content in the capsule (Figure 1b).

**Figure 2:**
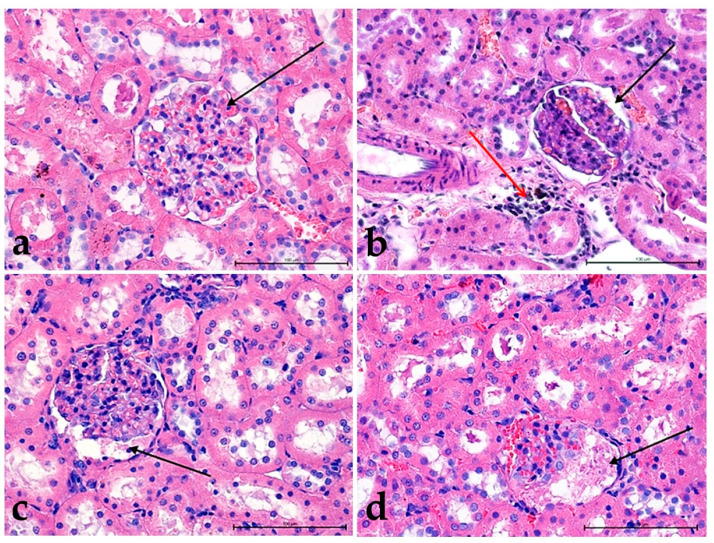
The changes in the structure of the kidneys in groups: **II** (**a**), **IIa** (**b**), **III** (**c**), **IIIa** (**d**). Glomeruli with different urine content (black arrow). Note the presence of leukocytes in the kidney parenchyma in **IIa** group (**b**) (red arrow). H&E staining. Mag. 400×. Bar scale 100 µm.

The results of morphological studies indicated in groups **IIa** (Figure 2b), **III** (Figure 2c), **IIIa** (Figure 2d) moderately increased urogenesis (visible intensive glomerular filtration), and slightly greater congestion were present in groups: **II** (Figure 2a), **III** (Figure 2c) and **IIIa** (Figure 2d). In rats fed with a high-cholesterol diet and watered the extract of *Ilex paraguariensis* (group **IIa**), there was some toxic effect in the kidney cortex, visible as the presence of proliferating fibroblasts and lymphocytes near large vessels (Figure 2b).

**Figure 3:**
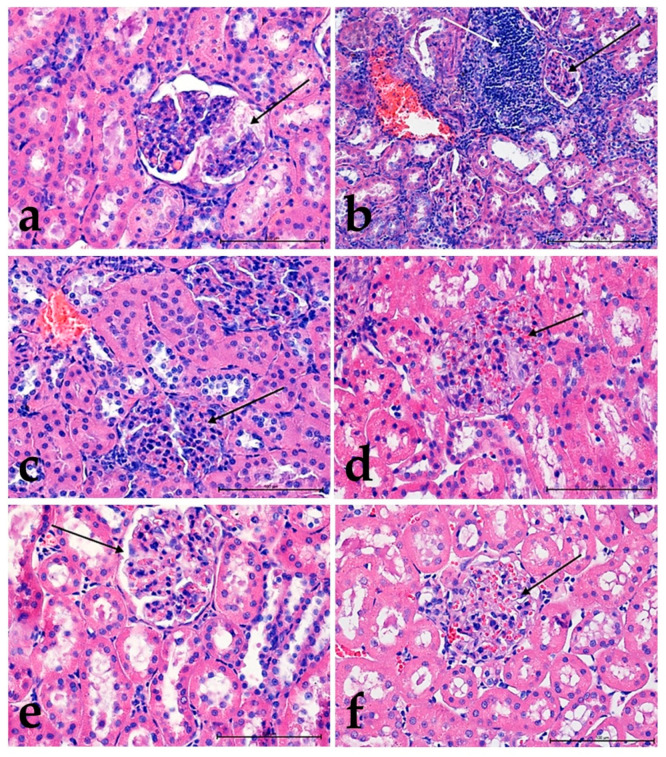
The changes in the structure of the kidneys in groups: **IV** (**a**), **IVa** (**b**), **V**(**c**), **Va** (**d**), **VI** (**e**), and **VIa** (**f**). A glomerulus with different urine and blood content (black arrow). The massive lymphocyte infiltration around the glomerulus in group **IVa** (**b**) (white arrow) H&E staining. Mag. 400×. Bar scale 100 µm.

Among the three fractions tested, the most potent activity enhancing urogenesis with numerous sites showing the arrested vascular flow and increased activity of connective tissue cells were observed in groups: **IV** (Figure 3a), **IVa** (Figure 3b), **Va** (Figure 3d), **VI** (Figure 3e) were the weakest in group **V** (Figure 3c). In group **VIa**, the outlook was similar, but it seems that the proximal and distal tubules were less filled with urine (Figure 3f). The toxic effect was observed as venous stasis with the presence of proliferating fibroblasts. Moreover, extensive lymphocyte infiltrates in large vessels’ vicinity were particularly noticeable in group **IVa** (Figure 3b).

### 3.3. Alcian Blue Staining

**Figure 4:**
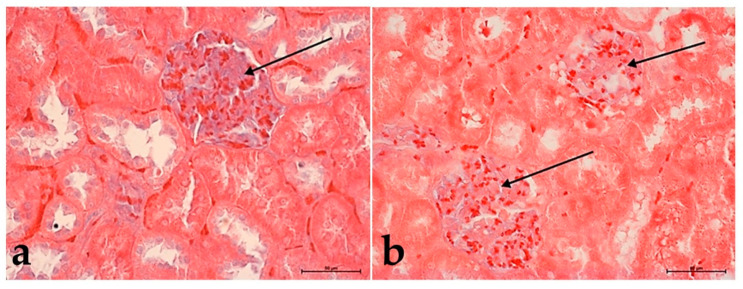
The changes in the kidneys’ structure in the controls group: in **I**, animals fed with normal (**a**) and **II**, high-cholesterol diet (**b**). Note the high content of proteoglycans (blue color) in the capillary tuft of the glomerulus (black arrow). Alcian blue staining. Mag. 200×. Bar scale 50 µm.

Morphological analysis of Alcian blue staining did not show significant differences in polysaccharides’ content within the glomeruli in control groups. In both groups, glomeruli were filled with numerous erythrocytes (Figure 4a,b).

**Figure 5:**
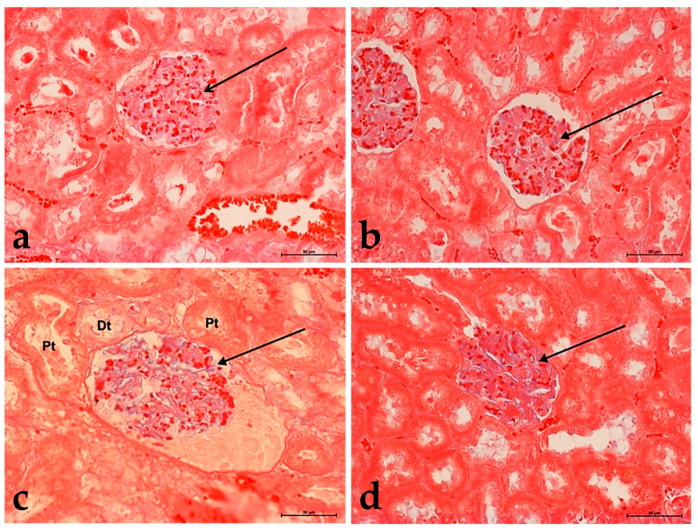
The changes in the structure of the kidneys in animals fed with a normal diet—groups **II** (**a**), **III** (**c**), and in animals fed with high-cholesterol diet—groups **IIa** (**b**), **IIIa** (**d**). A glomerulus with different proteoglycan content (arrow). Note the presence of urine in the glomerulus in group **III** (**c**). Alcian blue staining. Mag. 200×. Bar scale 50 µm.

**Figure 6:**
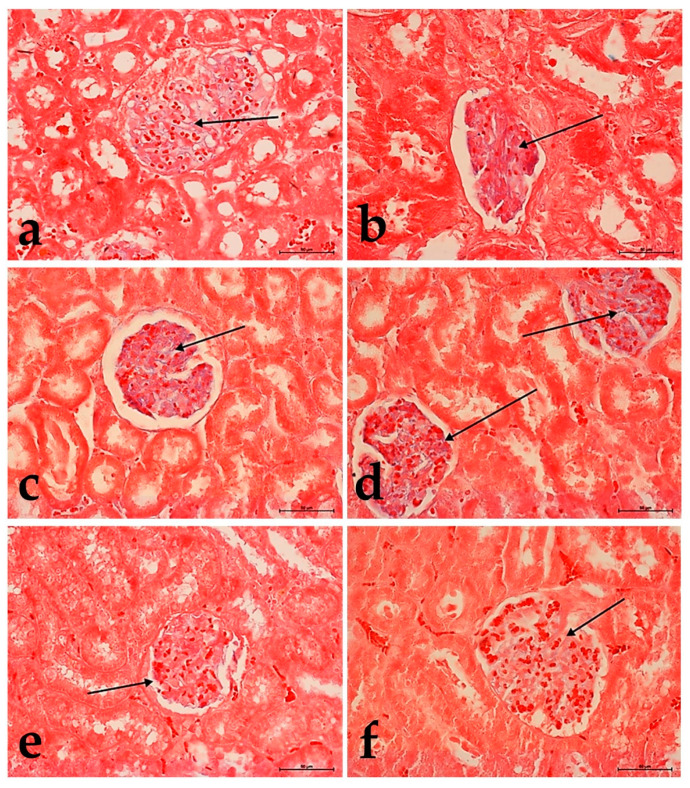
The changes in the proteoglycans content in the kidneys from groups **IV** (**a**), **V** (**c**), and **VI** (**e**) fed with normal diet and from groups **IVa** (**b**), **Va** (**d**), **VIa** (**f**) fed with a high-cholesterol diet. A glomerulus with different urine and blood content (black arrow). Noticeable differences in the lumen of proximal and distal tubules result from different levels of diuresis. Alcian blue staining. Mag. 400×, Bar scale 50 µm.

The tissue staining with Alcian blue showed that in groups **II** (Figure 5a), **III** (Figure 5c), **IV** (Figure 6a), and **V** (Figure 6c), the active compounds had an influence on the blood-urine barrier, leading to increased urogenesis. The study revealed that cholesterol significantly reduces this effect because the polysaccharide content decrease was observed only for group **VIa** (Figure 6f). In the other groups, no such differences were found (Figure 5b,d and Figure 6b).

### 3.4. Morphometric Analysis

The morphometric analysis of the first nutrient group showed a significant reduction in the content of basal membrane polysaccharides within the glomeruli in groups **II**, **III**, **IV**, and **V** fed with a normal diet. These observations were confirmed for the second group—fed with a high-cholesterol diet in the morphometric study. A slight decrease in the polysaccharide content was observed in group **VI**.

#### 3.4.1. Thickness of the Basement Membrane

**Figure 7:**
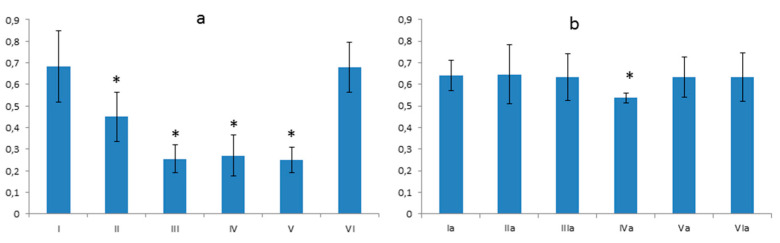
The membrane thickness of the capillary tuft. (**a**) Differences between the control group—**I** and groups **II**, **III**, **IV**, **V** were statistically significant at *p* = 0.05 (and signed by *); (**b**) Difference between the control group—**Ia** and **IVa** group was statistically significant at *p* = 0.05 (and signed by *). Vertical scale units expressed in µm.

The results of the morphometric analysis of the basement membrane’s thicknesses in the glomerulus showed that glycoprotein content in the glomerulus is different in subsequent groups (Figure 7). The differences between group **I** and groups **II**, **III**, **IV**, **V** were statistically significant (Figure 7a). The **I**st group in relation to **VI**th is negligible (Figure 7a). In the high cholesterol groups, the content of polysaccharides in the glomerulus was equal in most groups except the **IVa** group. The differences between the **Ia** and **IVa** groups were statistically significant (Figure 7b).

#### 3.4.2. Comparison of the Surface of the Glomerular Capsule to the Capillary Tuft

**Figure 8:**
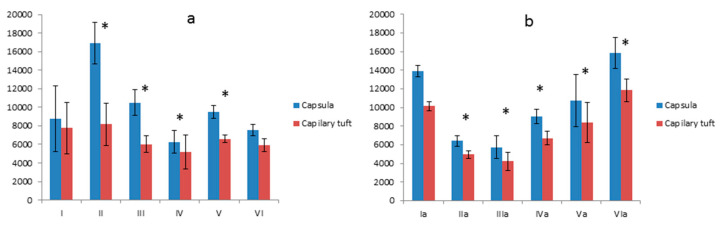
The capsule’s surface area and capillary tuft in kidneys of animals fed with regular diet (**a**) and fed with a high-cholesterol diet (**b**). Differences were statistically significant in respect to the control group at *p* = 0.05 (and signed by *). Vertical scale units expressed in µm.

In animals fed with a regular diet, there was an increase in glomerular capsule size in groups **II** and **III**. Minimal statistically significant growth was observed in group **V** and a decrease in group **IV**. There were no changes in group **VI** (Figure 8a). In the animals fed with a high-cholesterol diet, there was an apparent decrease of the glomerular surface in **IIa**, **IIIa**, **IVa**, and **Va** groups and a slight increase in group **VIa** (Figure 8b). A statistically significant differences compared to the control were reported in groups: **II**, **III**, **IV**, **V**, **IIa**, **IIIa**, **IVa**, **Va**, **VIa**.

#### 3.4.3. The Ratio between the Size of the Glomerular Capsule and the Capillary Tuft

**Figure 9:**
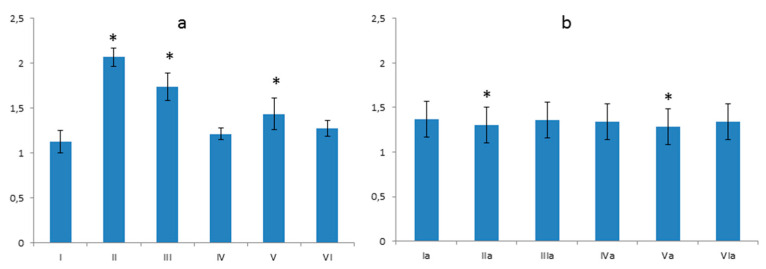
The ratio of the capsule surface to the capillary tuft surface in rats fed with regular diet (**a**). The ratio of the capsule surface to the capillary tuft surface in rats fed with a high-cholesterol diet (**b**). Differences were statistically significant in respect to the control group at *p* = 0.05 (and signed by *). Vertical scale units expressed in µm.

In this analysis, there is an increase of the glomerular surface capsule in groups: **II**, **III**, **V** in relation to group **I**. In groups **IV** and **VI**, the increases were not statistically significant (Figure 9a). In the second nutritional group, a decrease in this value is generally observed, which in the situation of the slightly increased surface area of group **Ia** indicates a somewhat normalizing effect in group **IIa** and **Va** (more potent) and contained in the components in groups **IIIa**, **IVa**, and **VIa** on vascular tuft (Figure 9b).

The detailed statistical differences concerning the performed measurements between all groups are presented in Appendix A (Table A4, Table A5, Table A6, Table A7, Table A8 and Table A9).

## 4. Discussion

The water extracts of *I. meservae* leaf contain: water-soluble microelements, carbohydrates and nitrogen-containing metabolites, polar phenolics (caffeoylquinic acids and glycosides of flavonoids), and saponins (glycosides of triterpenoids). Starting the work on testing the safety of prolonged administration of *I. meservae* leaf infusion in animals (rodents), we assumed to observe significant differences among the kidney-targeted effects of administration of standard infusion and the main, easy-separable groups of constituents of *Ilex* leaf (phenolics, saponins, and non-polar terpenoids). From the three assessed fractions, the positive role of polyphenols is widely described, whereas data about saponins as well as terpenoids are limited. However, together they seem to have numerous positive activities. In vivo studies conducted by de Oliveira et al. [20] indicate that *Ilex paraguariensis* plays an important, protective role in obesity and liver function. Peroral administration of water, ethanol, and refined *n*-butanol extracts from its leaves at levels 200, 400, and 800 mg/kg BW/day of (calc. on dry extract) during 30 days resulted in a decrease in serum triglycerides and cholesterol levels and a decrease in the atherosclerotic index in animal models [20]. The results also indicate the potential positive effect of this extract on the cardiovascular system [21]. Some of the pharmacological effects of *I. paraguariensis* are associated with a high content of caffeic acid (and its derivatives), flavonoids, and hydroxylated derivatives of cinnamic acid, which have antioxidant and anti-inflammatory activities [22]. Among the biological activities of Yerba Mate, it is essential to highlight its inhibitory effect on the enzymes involved in the initiation and maintenance of the inflammatory response [23]. Most of the reports concentrate on the positive effects of *I. paraguariensis,* but there are also reports on toxic properties of *Ilex* spp. on human and animal bodies. Therefore, according to de Andrade et al. [24], administration of acute and subchronic toxicity of Yerba Mate dose (2 g/kg body weight of Wistars rats) did not change the macroscopic and histopathological assessment of organs, including liver and kidneys. However, Kataoka et al. [25] found changes in kidneys after green tea administration in rats different from our research. They found that polyphenol intake during lactation by offsprings may play a protective role against a high-fat diet. They also found that polyphenols’ high content in a low-fat diet is causing inflammation and fibrosis in kidneys. According to our study, in group feed with polyphenols, increased inflammation in the kidney was associated with increased urination. One possible reason for the toxic effect of polyphenols is the high amounts of chlorogenic acid (CGA) among the polyphenols groups. Ae-Sim Cho et al. found that chlorogenic acid exhibits anti-obesity property and improves lipid metabolism in high-fat diet-induced obese mice [26]. Similar conclusions can be based on Metwally results [27]. This phenomenon’s possible mechanism is that CGA promotes fatty acid catabolism by regulating the AMPK pathway [28]. This positive feature of polyphenols in organisms is associated with reducing fat in blood plasma but can be a reason for kidney damage in a low-fat diet, especially at high doses; such correlation was noted by Murakami et al. [29]. Furthermore, the concentration of chlorogenic acid in tea is significantly lower than in Yerba Mate. The following phenolics were detected and counted previously in several European Ilex species and cultivars [12]: a pattern of mono- and dicaffeoylquinic acids accompanied by some flavonoids like quercetin 3-O-rutinoside, kaempferol 3-O-rutinoside, and unspecified quercetin 3-O-hexoside. According to Zwyrzykowska et al. [12], a sum of chlorogenic acids is comparable between *I. paraguariensis* and *I. meserveae* and reaches about 50% of total polyphenols. The differences are visible in dicaffeoyloquinic acid (40 vs. 11 rel%) and flavonoids (3 vs. 30 rel%). The results implicate that extracts should be used in pathological conditions but not in healthy animals. Interestingly, we found that polyphenols have light toxic action on kidneys and induce inflammation process; however, in both extracts (*I. paraguariensis* and *I. meserveae*). only in *I. paraguariensis* did we observe lymphocyte infiltration in the kidney stroma.

Plants of the genus *Ilex* are a rich source of saponins that may have antiproliferative effects and also exhibit anti-hypercholesterolemic, anti-parasitic, and anti-inflammatory effects [30,31]. It is possible that saponins, known to be able to induce cholestasis by liver destruction and act as irritants to the GI (gastrointestinal) tract, may also act synergistically or facilitate the intestinal absorption of co-administered substances and therefore induce additional negative effects on the organism. Another study also reports that *I. paraguariensis* is characterized by water-soluble polysaccharides, mainly arabinogalactans, which have a strong protective effect against gastritis [32]. Moreover, Dartora et al. showed in an animal study that oral administration of rhamnogalacturonan from the leaves of *I. paraguariensis* might be a promising adjuvant for sepsis treatment [33].

There is no comprehensive literature about the tested substances’ influence on the filtration barrier status. Initial histological analysis showed that depending on the diet used, the extracts from *I. paraguariensis* and *I. meserveae* are diuretic, and this is due to the different mechanisms of action of the individual components of the extracts. The intercellular content has a significant influence on selective permeability in renal filtration [34]. The glomerular basement membrane comprises proteoglycans—mainly heparan sulfate—and proteins—type IV collagen and laminins [5]. Heparan sulfate proteoglycans in the glomerular basal membrane (HSPGs) are a class of biomolecules with structural and regulatory functions. They are involved in biological processes such as glomerular filtration, cell adhesion, migration, proliferation, and differentiation. In response to numerous cytokines, proteoglycans are degraded by enzymes released by neighboring cells. Therefore, the content decreases during any inflammation process [35]. In our study, increased infiltration by leukocytes was noted in groups **II**, **III**, and **IVa**, and sporadically, single cells were found in other groups. To determine the changes occurring in the glomerulus, morphometric analysis of the size of the glomerular capsule and the capillary tuft was used, as well as the content of polysaccharides within the glomerulus. Our results suggest that increased urogenesis related to the use of *Ilex* extracts and fractions is associated with a decrease in proteoglycans content in the capillary tuft.

## 5. Conclusions

A standard animal model was used in this study, as well as the cholesterol dose that is usually used in this type of study. It was shown that the synergistic effect of polyphenols and terpenoids in a high-cholesterol diet can be used equally to protect the kidney. The effect of the biologically active compounds used can be determined in the future using a metabolic model, or an animal model of multifunctional aging. In the animal models proposed for the future, the compounds used could affect the already occurring changes in urogenesis, indicating a detrimental or protective effect. The applied dose of active compounds and friction could also be a limiting factor. However, the dose of extracts was applied according to the available literature [19].

Saponin fraction from *Ilex × meserveae* seems to have no influence on kidney status at the administered concentrations. However, polyphenols and terpenoids present in dry extracts and the fresh infusions from *Ilex × meserveae* and *Ilex paraguariensis* together with co-extracted substances in a normal diet cause a nephrotoxic effect which is decreased by a high-cholesterol diet. This synergistic effect can be used equally to protect the kidney against polyphenols and terpenoids.

## Figures and Tables

**Table 1:** Research groups of experimental animals. Control groups: **I**, **Ia**, and other groups with diet modified by the addition of *Ilex* extracts (**II**–**VI**) or both *Ilex* extracts and cholesterol (**IIa**–**VIa**).

Group	Diet Type (8 Animals in Each Diet Group)
**I**	rats fed with a standard diet
**Ia**	rats fed as a group **I** but with the addition of 20 g of cholesterol per kilogram of diet
**II**	rats receiving instead of drinking water the water extract of *I. paraguariensis* (each day, freshly infused extract was prepared by extraction of 50 g of leaves with 1L boiled water; every two animals had free access to 250 mL of this sole source of drink per day)
**IIa**	rats fed as group **II** but with the addition of 20 g of cholesterol per kilogram of diet
**III**	rats receiving instead of drinking water the water extract of *I. meserveae* “Blue Angel” (each day, freshly infused extract was prepared by extraction of 50 g of leaves with 1L boiled water; every two animals had free access to 250 mL of this sole source of drink per day)
**IIIa**	rats fed as group **III** but with the addition of 20 g of cholesterol per kilogram of diet
**IV**	rats receiving additionally polyphenol fraction from *I. meserveae* “Blue Angel” (each day, the dry extract was freshly solubilized in water in a dose of 10 mg/kg BW; every two animals had free access to 250 mL of this sole source of drink per day)
**IVa**	rats fed as group **IV** but with the addition of 20 g of cholesterol per kilogram of diet
**V**	rats receiving additionally terpenoid fraction from *I. meserveae* “Blue Angel” (each day, 200 mL of oil was mixed with terpenoids (in a dose 10 mg/kg BW) and 1 kg of feed and left overnight; every two animals had free access to diet and drinking water, supplied ad libitum as in group **I**)
**Va**	rats fed as group **V** but with the addition of 20 g of cholesterol per kilogram of diet
**VI**	rats receiving additionally saponin fraction from *I. meserveae* “Blue Angel” (each day, the dry extract was freshly solubilized in water in a dose of 10 mg/kg BW; every two animals had free access to 250 mL of this sole source of drink per day)
**VIa**	rats fed as group **VI** but with the addition of 20 g of cholesterol per kilogram of diet

## Data Availability

Not applicable.

## Appendix A

### Appendix A.1. Terpenoid Fraction

#### Appendix A.1.1. Preparation

To 100 g of dry, ground (<0.05 mm) leaves of *I. meserveae*, 350 mL of chloroform was added, cold-macerated for 24 h, and evaporated on a rotary evaporator. The residue was dissolved in 50 mL of hexane and washed three times with 50 mL of a methanol: water mixture (80:20 with 20 μL 2M hydrochloric acid 37%). After centrifugation and evaporation of hexane extract, the oily green residue was obtained. It was separated on a chromatography column (Kieselgel 60, 230–400 mesh, Merck) into the waxy- and triacylglycerol fractions (eluted with hexane:diethyl ether = 80:1) and the expected terpenoids (eluted with the same solvents in ratio 1:1). After preliminary identification (TLC and GC-MS), it was concentrated in vacuo to give 0.8 g of a white amorphous substance.

#### Appendix A.1.2. Analysis

The triterpenoid profile was evaluated using a N,O-*bis*-(trimethylsilyl)trifluoroacetamide (BSTFA) derivatization method. GC-MS Shimadzu QP 2020 (Shimadzu, Kyoto, Japan) was used for identification of constituents. 500 µL of pyridine and 50 µL of BSTFA were added to the dry sample (approximately 20 mg). The mixture was transferred to a vial and heated for 25 min at 70 °C. The separation was achieved using a Zebron ZB-5 capillary column (30 m, 0.25 mm, 0.25 μm; Phenomenex, Torrance, CA, USA). GC-MS analysis was performed according to the following parameters: scans were performed from 40 to 1050 *m*/*z* in electron impact ionization (EI) at 70 eV with a mode of 10 scans per second. Analyses were performed using helium as a carrier gas at a flow rate of 1.0 mL/min at split 1:20, and the following program: 100 °C for 1 min, 2.0 °C/min temperature rise from 100 to 190 °C; 5 °C/min temperature rise from 190 to 300 °C. The injector was maintained at 280 °C, respectively. Compounds were identified using two different analytical methods for comparison; retention times with authentic chemical compounds (Supelco C7-C40 Saturated Alkanes Standard) and mass spectra were obtained, with an available library (Willey NIST 17, fit index above 90%).

All triterpenoids were identified as corresponding trimethylsilylderivatives (TMS).

**Table A1:** GC-MS profile of triterpenoid fraction of *Ilex meserveae*.

KI exp.	KI lit.	RT [min]	Compound	*Ilex × meserveae* RA [%]
2835	2832	20.65	*all-trans*-Squalene	0.69
3162	3141	24.93	*a*-Tocopherol, TMS derivative	2.21
3361	3370	27.78	(3β)-Olean-18-en-3-ol (TMS)	tr
3370	3344	27.93	*β*-Sitosterol (TMS)	7.67
3384	3353	28.16	*β*-Amyrin (TMS)	9.05
3397	3385	28.37	Germanicol (TMS)	2.69
3420	3406	28.79	*α*-Amyrin (TMS)	28.97
3427	3435	28.93	Lupeol (TMS)	17.34
3508	3523	30.50	Lupeoyl acetate	0.75
3530	3540	30.99	Uvaol, 2-O-TMS	2.35
3563	3560	31.73	(3α)-Lup-20(29)-ene-3,28-diol (O,O-*bis*-TMS)	12.13
3580	3588	32.11	Betulinic acid (O,O-*bis*-TMS)	1.90
3596	3591	32.46	Oleanolic acid (TMS)	1.00
3643	3657	33.60	Ursolic acid (TMS)	5.22
3650	n.a.	33.75	Maslinic acid * (TMS)	6.54

KI exp.—retention index calculated according to linear *n*-alkanes; KI lit.—values of retention indices presented in NIST17 (national institute of standards and technology; Gaithersburg, MD, USA) database; RT—retention time; *—tentatively identified, only based on the mass spectrum, retention index is missed in the database; RA—relative area.

### Appendix A.2. Saponin Fraction

#### Appendix A.2.1. Preparation

60 g of lyophilized *Ilex* × *meserveae* “Blue Angel” leaves powder was 1-day macerated at room temperature, two times, each with 600 mL 70% methanol. After that, the extracts were combined, filtered through a filter paper, and the solution of 30 g lead (II) acetate trihydrate in 70% methanol (in a total amount of 100 mL) was added to precipitate ballast substances (e.g., phenolics). The extract was centrifuged at 3000 rpm for 15 min (MPW). The residue containing complexes of ballast substances was subjected to an experimental and safe process of its utilization by Paweł Lochyński., Wrocław University of Science and Technology. The possible residues of lead ions in the saponin-rich fraction were precipitated with the addition of diluted sulphuric acid, centrifugation, and further neutralization of the supernatant.

The supernatant was diluted with distilled water to obtain 40% methanol concentration and centrifuged again. Afterward, the second supernatant was applied to a 20 g octadecyl SPE column (J. T. Baker) in two runs. The saponin enriched fraction was recovered from the SPE bed by a minimal volume of pure methanol. Combined saponin fractions from SPE were concentrated with a vacuum evaporator (Büchi) at 40 °C to dryness, and the glassy residue was lyophilized.

One reference Holly (*Ilex aquifolium*) and two reference Mate (*Ilex paraguariensis*) were prepared similarly to *I. meserveae* saponin fraction (Il.6) on an analytical scale. A measure of 100 mg of each plant substance was macerated in 2 mL of 70% methanol for 15 min. in an ultrasonic bath (Bandelin). After that, the solution of 0.05 g lead (II) acetate trihydrate in 70% methanol (in a total amount of 0.5 mL) was added to precipitate ballast substances. The extracts were further treated as described above.

#### Appendix A.2.2. UHPLC-ESI-MS Conditions of Analysis of Saponins

The UHPLC Ultimate 3000 apparatus (Thermo Fisher Sci.) consisted of a quaternary pump with a vacuum degasser, autosampler, and a thermostated column chamber combined with an ESI-qTOF Compact detector (Bruker Daltonics).

Separations were achieved on Kinetex C-18 (150 × 2.1 mm, 2.6 μm; Phenomenex) with a flow rate of 0.3 mL/min and column temperature of 30 °C. The elution system consisted of the following gradient steps of water (A) and acetonitrile (B) with 0.1% addition of formic acid: 0→1 min. (2→30% B in A), 1→31 min. (30→60% B in A), 31→31.5 min. (60→100% B in A), 31.5→35.5 min. (100% B in A). The extracts were diluted with acetonitrile/water (1:1, *v*/*v*) to obtain the final concentrations of 0.02 mg per mL; the injection volumes were 5 μL.

The detector worked in negative mode and was calibrated with the Tunemix^TM^ (Bruker Daltonics) with *m*/*z* SD below 1 ppm. A calibration segment was at the beginning of every single run. The mass accuracy of main saponins found in reference Mate extract and verified with literature was within 3 ppm. The main instrumental parameters were as follows: scan range 50–2200 *m*/*z*, nebulizer pressure 1.5 bar, dry gas—nitrogen—7.0 L/min, temperature 200 °C, capillary voltage 2.2 kV, ion energy 5 eV, collision energy 10 eV vs. 30 eV, low mass set at 200 *m*/*z*. The analysis of the obtained mass spectra was carried out using a Data Analysis (Bruker Daltonics).

#### Appendix A.2.3. Results of UHPLC-ESI-MS Saponin Profiling

Because no information on saponin profile in *I. meserveae* was found in the literature, its saponin profile was compared with commercially available Mate (*I. paraguariensis*) and holly (*I. aquifolium*). With the information that *I. meserveae* is suspected to be a hybrid of *I. rugosa* and *I. aquifolium* or *I. rugosa* and *I. cornuta*, it was expected that similar compounds as in the mother plant could be found in Il.6.

Comparing saponin profiles of *I. meserveae* and *I. paraguariensis* led to the observation that their pattern in *I. meserveae* is much less complicated than in commercial Mate, as shown in Table A2. In this case, compounds **1**–**4** were of similar relative intensities. Five of them (3–5, 9, 13) were also found in Mate with different concentrations. The most intensive peaks belonged to substance 3 (of molecular weight (MW) 1074 Da) and substance 5 (MW 928 Da), which was found in both Yerba Mate samples on the level of about 10–70% and 9–19% of the relative area (RA). Substances 1 and 2 were absent in the analyzed Yerba Mate.

As we do not possess reference saponins from Mate, we can speculate (by relative retention and accurate molecular mass measurements) that peak 18, the most intensive one in Yerba Mate, could be matesaponin 1 (MW 912 Da), peak 16 or 17 could be matesaponin 2 (MW 1058 Da), peak 3 or 4 could be matesaponin 3 (MW 1074 Da), peak 9 could be matesaponin 4 (MW 1220 Da), and peak 6 could be matesaponin 5 (MW 1382 Da). Chemical Abstracts search for substance 1 revealed five isomeric saponins (MW 1236 Da) isolated from genus *Ilex*, while for substance 2, six isomeric saponins (MW 1074 Da) isolated from the genus *Ilex*. That initial finding showed that isolation and NMR verification of each structure would be necessary to define the substances precisely if needed. However, some additional assumptions were made using interpretations included in a paper of Negrin and co-workers [36].

**Table A2:** UHPLC-ESI-MS comparison of main saponin profiles of *I. meserveae* saponin-rich fraction (Il.6) and reference commercial Mate samples (Il.1a and Il.1b).

No.	Compound Number Reported in [36]	[M–H]^−^ Measured [*m*/*z*]	Proposed Formula	Il.1a		Il.1b		Il.6	
				RT [min]	RA [%]	RT [min]	RA [%]	RT [min]	RA [%]
1.	—	1235.61	C_59_H_96_O_27_	—	—	—	—	6.78	73.40
2.	**39** ^a^	1073.56	C_53_H_86_O_22_	—	—	—	—	7.40	70.53
3.	**39** ^a^	1073.56	C_53_H_86_O_22_	8.07	60.97	8.03	10.70	8.06	100.00
4.	**39** ^a^	1073.56	C_53_H_86_O_22_	8.10	20.01	8.07	5.07	8.10	11.53
5.	**40**	927.50	C_47_H_76_O_18_	8.24	18.63	8.22	8.24	8.26	99.22
6.	**41**	1381.68	C_65_H_106_O_31_	8.48	11.11	8.47	2.78	—	—
7.	**43**	1131.56	C_55_H_88_O_24_	8.70	10.70	—	—	—	—
8.	**45**	1101.55	C_54_H_86_O_23_	8.85	14.20	—	—	—	—
9.	**47**	1219.61	C_59_H_96_O_26_	9.10	59.09	9.11		9.11	13.99
9a.	**48**	911.50	C_47_H_76_O_17_	9.17	5.10	—	—	9.21	31.68
10.	**49**	927.50	C_47_H_76_O_18_	9.45	18.28	9.42	8.86	9.45	10.62
11.	**56** ^b^	1235.58	C_62_H_92_O_25_	10.13	4.05	—	—	—	—
12.	**56** ^b^	1235.58	C_62_H_92_O_25_	10.40	9.40	—	—	—	—
13.	**55**	1057.56	C_53_H_86_O_21_	10.78	27.96	10.72	11.11	10.82	48.36
14.	**56** ^b^	1235.58	C_62_H_92_O_25_	11.08	4.35	—	—	—	—
15.	**57**	1115.57	C_55_H_88_O_23_	11.23	20.73	11.17	7.61	—	—
16.	**62**	1057.56	C_53_H_86_O_21_	11.85	100.00	11.78	94.64	—	—
17.	**64**	1057.56	C_53_H_86_O_21_	12.09	68.77	12.04	54.22	—	—
18.	**66** ^c^	911.50	C_47_H_76_O_17_	12.49	93.17	12.44	100.00	—	—
19.	**66** ^c^	911.50	C_47_H_76_O_17_	12.68	11.98	12.63	7.92	—	—
20.	**66** ^c^	911.50	C_47_H_76_O_17_	12.91	20.45	12.85	13.00	—	—
21.	**72**	1085.56	C_54_H_86_O_22_	13.63	8.95	13.56	2.80	—	—
22.	**77**	895.51	C_47_H_76_O_16_	14.34	20.19	14.28	18.10	—	—
23.	**80**	895.51	C_47_H_76_O_16_	14.63	6.43	14.55	5.83	—	—
24.	**83**	953.52	C_49_H_78_O_18_	14.86	30.53	14.77	25.58	—	—
25.	**82** ^d^	1219.59	C_59_H_96_O_26_	16.24	3.25	16.21	3.55	—	—
26.	**82** ^d^	1219.59	C_59_H_96_O_26_	16.54	2.84	16.53	2.26	—	—
27.	**108** ^e^	895.51	C_47_H_76_O_16_	20.36	3.68	20.36	4.03	—	—
28.	**108** ^e^	895.51	C_47_H_76_O_16_	20.50	2.34	20.50	1.83	—	—
29.	**110**	749.45	C_41_H_66_O_12_	22.18	3.33	22.15	3.42	—	—

RT—retention time, RA—the relative area of peak, when the area of the largest one is calculated as 100%. The same letters in the column referring to [36] means the same possible assignments of detected compounds.

**Table A3:** Database- and MS/MS-based identification of main saponins present in *Ilex paraguariensis* (Il.1), *I. aquifolium* (Il.5), and *I. meserveae* (Il.6).

Compound	Source	MS/MS Interpretation	Probable Identification
**1** Rt = 6.8 min; calc. [M–H]^–^ = 1235.6061, err. 0.5 ppm; neutral formula: C_59_H_96_O_27_	Il.5 Il.6	911 [M–(Hex+Hex)–H]^–^ 765 [M–(Hex+Hex)–dxHex–H]^–^ 749 [M–(Hex+Hex)–Hex–H]^–^ 731 [M–(Hex+Hex)–Hex–18–H]^–^ 603 [M–(Hex+Hex)–Hex–dxHex–H]^–^ 471 [M–(Hex+Hex)–Hex–(dxHex+Pen)–H]^–^	kudinoside N (SA)
**2** Rt = 7.4 min; calc. [M–H]^–^ = 1073.5538, err. −0.1 ppm; neutral formula: C_53_H_86_O_22_	Il.6	749 [M–(Hex+Hex)–H]^–^ 731 [M–(Hex+Hex)–18–H]^–^ 453 [M–(Hex+Hex+18)–(dxHex+Pen)–H]^–^	latifoloside L (PA) matesaponin 3 (UA)
**3/4** Rt = 8.0 min/8.1 min calc. [M–H]^–^ = 1073.5538, err. −0.3 ppm; neutral formula: C_53_H_86_O_22_	Il.1 Il.5 Il.6	911 [M–Hex–H]^–^ 765 [M–Hex–dxHex–H]^–^ 749 [M–Hex–Hex–H]^–^ 731 [M–Hex–Hex–18–H]^–^ 603 [M–Hex–(Hex+dxHex)–H]^–^ 471 [M–Hex–(Hex+dxHex)–Pen–H]^–^	latifoloside L (PA) matesaponin 3 (UA)
**5** Rt = 8.3 min; calc. [M–H]^–^ = 927.4959, err. 0.2 ppm; neutral formula: C_47_H_76_O_18_	Il.1 Il.5 Il.6	765 [M–Hex–H]^–^ 603 [M–Hex–Hex–H]^–^ 471 [M–Hex–Hex–Pen–H]^–^	ilexoside XV (SA) ilexoside II (PA) ilekudinoside E (PA) ilexsaponin B3 (IG-B)
**9** Rt = 9.1 min; calc. [M–H]^–^ = 1219.6117, err. –0.2 ppm; neutral formula: C_59_H_96_O_26_	Il.1 Il.5 Il.6	895 [M–(Hex+Hex)–H]^–^ 749 [M–(Hex+Hex)–dxHex–H]^–^ 733 [M–(Hex+Hex)–Hex–H]^–^ 715 [M–(Hex+Hex)–Hex–18–H]^–^ 587 [M–(Hex+Hex)–(Hex+dxHex)–H]^–^ 569 [M–(Hex+Hex)–(Hex+dxHex)–18–H]^–^ 455 [M–(Hex+Hex)–(Hex+dxHex)–Pen–H]^–^	matesaponin 4 (UA)
**13** RT = 10.8 min; calc. [M–H]^–^ = 1057.5589, err. 0.1 ppm; neutral formula: C_53_H_86_O_21_	Il.1 Il.5 Il.6	733 [M–(Hex+Hex)–H]^–^ 587 [M–(Hex+Hex)–dxHex–H]^–^ 455 [M–(Hex+Hex)–dxHex–Pen–H]^–^	matesaponin 2 (UA) or isomer
**16** RT = 11.85 min; calc. [M–H]^–^ = 1057.5589, err. 0.5 ppm; neutral formula: C_53_H_86_O_21_	Il.1	895 [M–Hex–H]^–^ 749 [M–Hex–dxHex–H]^–^ 733 [M–Hex–Hex–H]^–^ 715 [M–Hex–Hex–18–H]^–^ 587 [M–Hex–(Hex+dxHex)–H]^–^ 569 [M–Hex–(Hex+dxHex)–18–H]^–^ 455 [M–Hex–(Hex+dxHex)–Pen–H]^–^	matesaponin 2 (UA) ilekudinoside A (OA)
**17** RT = 12.09 min; calc. [M–H]^–^ = 1057.5589, err. –0.5 ppm; neutral formula: C_53_H_86_O_21_	Il.1	895 [M–Hex–H]^–^ 749 [M–Hex–dxHex–H]^–^ 733 [M–Hex–Hex–H]^–^ 715 [M–Hex–Hex–18–H]^–^ 587 [M–Hex–(Hex+dxHex)–H]^–^ 569 [M–Hex–(Hex+dxHex)–18–H]^–^ 455 [M–Hex–Hex–dxHex–Pen–H]^–^	ilekudinoside A (OA)
**18** RT = 12.49 min; calc. [M–H]^–^ = 911.5010, err. –1.6 ppm; neutral formula: C_47_H_76_O_17_	Il.1	749 [M–Hex–H]^–^ 587 [M–Hex–Hex–H]^–^ 569 [M–Hex–Hex–18–H]^–^ 455 [M–Hex–Hex–Pen–H]^–^	matesaponin 1 (UA) guaiacin B (OA)
**24** RT = 14.86min; calc. [M–H]^–^ = 953.5115, err. –1.1 ppm; neutral formula: C_49_H_78_O_18_	Il.1	749 [M–Hex(Ac)–H]^–^ 731 [M–Hex(Ac)–18–H]^–^ 629 [M–Hex–Hex–H]^–^ 587 [M–Hex(Ac)–Hex–H]^–^ 569 [M–Hex(Ac)–Hex–18–H]^–^ 455 [M–Hex–Hex–Pen–H]^–^	*I. amara* saponin (UA), 77-52-1 *I. amara* saponin (OA), 508-02-1

Probable identification based on (1) saponin database of *Aquifoliaceae*, (2) MS/MS fragmentation pathway. Aglycones: IG-B—ilexgenin B, OA—oleanolic acid, PA—pomolic acid, SA—siaresinolic acid, UA—ursolic acid. MS/MS loss of sugar unit: dxHex—deoxyhexose, Hex—hexose, Hex(Ac)—acetylhexose, Pen—pentose.

**Table A4:** The membrane thickness of capillary tuft in rats fed with normal diet. Statistical differences between all experimental groups; * significant, 0 insignificant.

Group No	I	II	III	IV	V	VI
**I**	-	*	*	*	*	0
**II**	*	-	*	*	*	*
**III**	*	*	-	0	0	*
**IV**	*	*	0	-	0	*
**V**	*	*	0	0	-	*
**VI**	0	*	*	*	*	-

**Table A5:** The membrane thickness of capillary tuft in rats fed with high cholesterol diet. Statistical differences between all experimental groups; * significant, 0 insignificant.

Group No	Ia	IIa	IIIa	IV	Va	VIa
**Ia**	-	0	0	*	0	0
**IIa**	0	-	0	*	0	0
**IIIa**	0	0	-	*	0	0
**IVa**	*	*	*	-	*	*
**V**	0	0	0	*	-	0
**VIa**	0	0	0	*	0	-

**Table A6:** The capsule’s surface area and capillary tuft in kidneys of animals fed with regular diet. Statistical differences between all experimental groups; * significant, 0 insignificant.

Group No	I	II	III	IV	V	VI
**I**	-	*	*	*	*	0
**II**	*	-	*	*	*	*
**III**	*	*	-	*	0	*
**IV**	*	*	*	-	*	*
**V**	*	*	0	*	-	*
**VI**	0	*	*	*	*	-

**Table A7:** The capsule’s surface area and capillary tuft in kidneys of animals in high cholesterol diet. Statistical differences between all experimental groups; * significant, 0 insignificant.

Group No	Ia	IIa	IIIa	IV	Va	VIa
**Ia**	-	*	*	*	*	*
**IIa**	*	-	0	*	*	*
**IIIa**	*	0	-	*	*	*
**IVa**	*	*	*	-	0	*
**V**	*	*	*	0	-	*
**VIa**	*	*	*	*	*	-

**Table A8:** The ratio of the capsule surface to the capillary tuft surface in rats fed with regular diet. Statistical differences between all experimental groups; * significant, 0 insignificant.

Group No	I	II	III	IV	V	VI
**I**	-	*	*	0	*	0
**II**	*	-	*	*	*	*
**III**	*	*	-	*	*	*
**IV**	0	*	*	-	*	0
**V**	*	*	*	*	-	0
**VI**	0	*	*	0	0	-

**Table A9:** The ratio of the capsule surface to the capillary tuft surface in rats fed with a high-cholesterol diet. Statistical differences between all experimental groups; * significant, 0 insignificant.

Group No	Ia	IIa	IIIa	IV	Va	VIa
**Ia**	-	*	0	0	*	0
**IIa**	*	-	*	*	0	*
**IIIa**	0	*	-	0	*	0
**IVa**	0	*	0	-	*	0
**V**	*	0	*	*	-	*
**VIa**	0	*	0	0	*	-
